# Transcriptomic and Meat Quality Differences in Longissimus Dorsi Muscle of Surgically Castrated Three-Year-Old Kazakh Horses

**DOI:** 10.3390/biology15120959

**Published:** 2026-06-18

**Authors:** Zexu Li, Wanlu Ren, Ran Wang, Luling Li, Shikun Ma, Yi Su, Dehaxi Shan, Qiuping Huang, Jianwen Wang

**Affiliations:** 1 College of Animal Science, Xinjiang Agricultural University, Urumqi 830052, China; 2 Xinjiang Key Laboratory of Equine Breeding and Exercise Physiology, Urumqi 830052, China

**Keywords:** histomorphology, meat quality traits, surgical castration, transcriptomics

## Abstract

Although the Kazakh horse is a dual-purpose breed renowned for both milk and meat production, the extent to which surgical castration alters gene expression in its muscles has not yet been fully elucidated. In this study, left longissimus dorsi muscle (LDM) samples were obtained from six Kazakh stallions (W group) and six Kazakh geldings (S group) to comparatively evaluate meat quality parameters, examine histological characteristics in tissue sections, and apply transcriptomic profiling to comprehensively explore the principal regulatory pathways and candidate genes through which surgical castration modulates LDM growth. The results demonstrated that surgical castration did not induce significant alterations in meat color or pH-related parameters. However, cooking loss and shear force values were markedly diminished, accompanied by a marked decrease in muscle fiber cross-sectional area. Transcriptomic analysis identified 848 differentially expressed genes (DEGs) in total, comprising 415 upregulated and 433 markedly downregulated DEGs, which were predominantly enriched in key biological pathways, including actin cytoskeleton regulation. Moreover, eleven core candidate genes, including MYL2, MYL3, and TNNI1, were further screened and identified.

## Simple Summary

Although the Kazakh horse is a superior milk–meat dual-purpose breed, the mechanism by which surgical castration affects its muscle traits remains poorly understood. We analyzed the meat quality, histology and transcriptome of longissimus dorsi muscle from stallions and geldings. Castration did not alter meat color and pH, but decreased cooking loss, shear force and muscle fiber area. We detected 848 differentially expressed genes enriched in the regulation of the actin cytoskeleton, with 11 core genes, including MYL2, identified. This study reveals the mechanism of castration that improves meat quality in Kazakh horses, provides a theoretical basis for meat quality improvement and scientific breeding, and promotes the high-quality development of the horse meat industry.

## 1. Introduction

Kazakh horses exhibit distinctive germplasm attributes and are fully adapted to local climatic conditions as well as extensive feeding management systems. The meat produced is characterized by a firm texture and pronounced flavor, thereby satisfying prevailing market consumption requirements. This breed constitutes a valuable genetic resource that supports both quality enhancement and efficiency improvement within the horse meat industry, while integrating ecological adaptability with substantial economic value [1,2]. Nevertheless, compared with Yili horses, Thoroughbreds, Mongolian horses, and other mainstream equine breeds, there still exist obvious research gaps in the molecular mechanism governing meat quality formation, key regulatory genes related to muscle fiber development and fatty acid metabolism, as well as genetic differentiation among different ecotypes of Kazakh horses. Existing studies have preliminarily explored the genetic diversity and population structure of native Xinjiang horses, but systematic analysis on functional genes associated with Kazakh horsemeat quality is insufficient, especially in transcriptome-based screening of DEGs and exploration of regulatory pathways.

Conventional meat quality parameters (color, pH, cooking loss, and shear force) are well-established determinants of consumer preference [3,4,5,6]. However, these parameters alone cannot explain the structural basis of meat quality variation. Histomorphology enables direct observation of muscle fiber architecture (e.g., cross-sectional area, density), offering a mechanistic link between cellular structure and meat tenderness [7,8]—a link that remains unexplored in castrated Kazakh horses.

Skeletal muscle growth and development are complex biological processes regulated by multiple genes and signaling pathways, involving myocyte proliferation, differentiation, and fusion [9,10]. Transcriptome sequencing is a powerful approach for investigating the molecular changes in skeletal muscle induced by surgical castration. Studies in pigs and other livestock species have demonstrated that androgen deficiency alters muscle fiber characteristics and protein metabolism by regulating the expression of functional genes, while non-coding RNA interaction networks also contribute to castration-induced physiological changes in muscle [11,12,13]. However, most existing studies have focused on conventional livestock species, and the molecular mechanisms underlying the effects of castration on muscle growth and meat quality in Kazakh horses remain largely unclear. Therefore, this study employed transcriptome sequencing to characterize gene expression profiles in the skeletal muscle of castrated Kazakh horses, providing a foundation for elucidating the mechanisms of muscle development and meat quality formation in this indigenous horse breed.

This study was designed to evaluate the effects of surgical castration on meat quality traits, muscle fiber morphology, and transcriptomic profiles of the longissimus dorsi muscle (LDM) in three-year-old Kazakh horses, with the objective of identifying key candidate genes and regulatory pathways associated with muscle development and meat quality.

## 2. Materials and Methods

### 2.1. Animals and Sample Preparation

A total of 12 three-year-old Kazakh horses were included in this experiment, comprising six geldings and six stallions, with the geldings having undergone surgical castration at approximately two and a half years of age. All experimental Kazakh horses were raised at Qiaheji Pasture in Emin County, Tacheng Prefecture, Xinjiang Uygur Autonomous Region, China, under a unified feeding and management regime. The horses were fed high-quality alfalfa hay and corn pellets, with free access to drinking water, and their body weights were maintained at a similar level. Fresh longissimus dorsi muscle samples were collected at Wantong Livestock Slaughterhouse, Emin County, Tacheng Prefecture, Xinjiang. Muscle specimens were uniformly obtained 3 cm lateral to the transverse process of the 13th thoracic vertebra. All slaughter procedures for Kazakh horses were strictly conducted in accordance with international standards for equine slaughter [14]. After slaughter, LDM samples from Kazakh horses were collected, and two aliquots were placed into 5 mL cryopreservation tubes and stored at −80 °C for subsequent analysis. Additionally, two other portions were prepared: one sample was placed into a 50 mL cryopreservation tube and fixed with 4% paraformaldehyde, whereas the other was preserved in liquid nitrogen.

### 2.2. Meat Quality Measurements

Meat quality evaluation included measurements of muscle pH, SF, color, and CL.

1. pH value: Muscle pH was determined using a PH-818M portable pH meter (Wan Chuang Electronics MFG. Co., Ltd., Dongguan, China). The detailed measurement protocol followed the methodology outlined by Dorleku et al. [15].
2. CL: Approximately 100 g of raw meat was weighed. heated in a water bath at 80 °C until the geometric center of the sample reached 80 °C, as monitored by a RTS-209 thermocouple probe (Dongguan Chuanglian Electronic Technology Co., Ltd., Shenzhen, China). After cooling to room temperature, the sample was reweighed. Each sample was measured in triplicate, and the average value was calculated. The cooking loss was computed as follows: Cooking loss% = [starting weights (g) − cooked weights (g) × 100]/starting weight (g). All procedures were performed in accordance with the experimental protocol described by Al-Ghamdi et al. [16].
3. SF: Meat samples were cut into strips of 0.5 cm in thickness, 3 cm in length, and 0.5 cm in width along the direction of muscle fibers. Tenderness was determined using a C-LM3B muscle tenderness meter (Tenovo International Co., Ltd., Beijing, China). Each sample was measured nine times, and the average value was recorded. All procedures were performed in accordance with the experimental protocol described by Al-Ghamdi et al. [16].
4. Color: Meat color was measured using a SC-10 colorimeter (3nh Technology Co., Ltd., Guangdong, China). The instrument was calibrated with standard black and white plates before measurement. Each sample was measured in triplicate and the average value was recorded. The measurement was performed according to the method described by Qiu et al. [17].

### 2.3. Histomorphological Observation

The LDM tissue samples fixed in paraformaldehyde were retrieved and sectioned along the muscle fiber orientation into blocks with approximate dimensions of 0.2 cm in thickness, 0.5 cm in height, and 0.5 cm in width. The resulting blocks were placed into single-hole embedding cassettes and rinsed under running water for 12–16 h. Subsequently, paraffin sections were prepared in accordance with standard laboratory procedures for skeletal muscle histological analysis [18]. The paraffin sections underwent staining with the G1120 Hematoxylin and Eosin (H&E) Staining Kit (Beijing Solarbio Science & Technology Co., Ltd., Beijing, China). After staining, the sections were mounted with neutral resin and allowed to dry. The tissue slices were photographed using a light microscope (Eclipse E100 Nikon, Nikon Corporation, Tokyo, Japan) connected to a camera system. ImageJ (1.54f) software was used to perform quantitative morphological analysis of muscle fibers in LDM tissue sections. Indicators measured included muscle fiber diameter, muscle fiber perimeter, cross-sectional area, and muscle fiber density. The obtained data were used for subsequent statistical analysis. Three fields of view were randomly selected for each section, and all muscle fibers within these fields were measured. Approximately 80 muscle fibers were counted and measured per section in total, and the mean values were calculated as the final statistical results. Muscle fiber density was calculated as follows:

Density = number/area

### 2.4. Transcriptome Data Analysis

#### 2.4.1. Transcriptomic Detection and Data Analysis

RNA integrity and total RNA quantity were accurately assessed utilizing an Agilent 2100 bioanalyzer (Agilent Technologies, Santa Clara, CA, USA). RNA sequencing was executed on the Illumina platform, with sequencing services supplied by Novogene Co., Ltd. (Beijing, China).

Raw data underwent processing through Fastp (v0.23.4) software to produce high-quality clean reads. Reference genome index construction was executed using HISAT2 v2.0.5, followed by alignment of paired-end clean reads to the reference genome via HISAT2 v2.0.5. Novel gene prediction was carried out using StringTie (1.3.3b). FeatureCounts (1.5.0-p3) was utilized to determine the number of reads mapped to each gene, following which FPKM values for each gene were computed based on gene length and corresponding mapped read counts.

Differential expression analysis comparing two conditions/groups was performed utilizing the DESeq2R package (1.20.0). The resulting *p*-values underwent adjustment through the Benjamini and Hochberg method for false discovery rate control. Significant differential expression was determined using criteria of corrected *p*-value ≤ 0.05 and |log2 (fold change)| ≥ 0. Gene Ontology (GO) enrichment analysis of differentially expressed genes (DEGs), along with statistical enrichment analysis of DEGs in Kyoto Encyclopedia of Genes and Genomes (KEGG) pathways, was executed using clusterProfiler (3.8.1) software.

#### 2.4.2. Quantitative Real-Time Polymerase Chain Reaction (qRT-PCR) Validation

Total RNA extraction from 12 muscle samples was performed utilizing the Trizol method, employing the subsequent procedures. Briefly, 100 mg of tissue was placed into a grinding tube containing 1 mL Trizol and grinding beads, placed in a jxfstprp-32 grinder (Jingxin, Shanghai, China), and homogenized six times at a frequency of 60 Hz for 60 s per cycle. After complete homogenization, samples were permitted to rest for 10 min before centrifugation at 12,000 rpm for 10 min at 4 °C. The supernatant was harvested, and chloroform (0.2 mL per 1 mL Trizol) was incorporated, followed by vortex mixing for 15 s, room temperature incubation for 3 min, and centrifugation at 12,000 rpm for 15 min (with deceleration to 0). The obtained supernatant was transferred to a fresh centrifuge tube, and pre-chilled isopropanol (0.5 mL per 1 mL Trizol) was introduced. After vortex mixing for 15 s, the mixture underwent incubation at −20 °C for 30 min before centrifugation at 12,000 rpm for 10 min at 4 °C. The supernatant was discarded, and the RNA pellet underwent washing with 1 mL of 75% ethanol, followed by centrifugation at 7500 rpm for 5 min at 4 °C. The supernatant was eliminated, and the washing procedure was repeated once more. The RNA pellet was air-dried for approximately 10 min and reconstituted in an appropriate volume of diethyl pyrocarbonate-treated water. RNA concentration was then quantified using a NanoPhotometer^®^ N50 (Implen GmbH, Munich, Germany). Reverse transcription PCR was conducted for cDNA synthesis utilizing the PrimeScript™ RT reagent kit with gDNA Eraser (TaKaRa Bio, Shanghai, China). The thermal cycling parameters included 42 °C for 2 min, followed by 37 °C for 15 min, and 85 °C for 5 s. Upon completion of extraction and reverse transcription, RNA and cDNA samples were maintained at −80 °C until subsequent analysis. qRT-PCR was conducted using the QuantiNova SYBR PCR mix kit (QIAGEN, Shanghai, China) per the supplier’s protocols on a CFX96™ Real-Time System (Bio-Rad, Laboratories, Hercules, CA, USA). The thermal cycling protocol included an initial denaturation at 95 °C for 2 min, followed by 39 cycles of 95 °C for 2 s, annealing for 10 s, 65 °C for 5 s, and a final denaturation at 95 °C for 5 s. Quantitative data were evaluated using the 2^−ΔΔCt^ method. Glyceraldehyde-3-phosphate dehydrogenase served as the internal reference gene. Primer details are presented in Table 1.

### 2.5. Data Analysis

During the phenotypic data analysis phase of this investigation, data input and initial organization were conducted using Excel spreadsheet software. SPSS 26.0 (IBM Corporation, Armonk, NY, USA) was employed for differential analysis and correlation analysis. Prior to parametric tests, the normality of the data was assessed using the Shapiro–Wilk test, and homogeneity of variances was verified using Levene’s test. As the assumptions for parametric tests were met, an independent-sample *t*-test was used for differential analysis, and Pearson correlation analysis was used for correlation analysis. Given the exploratory nature of this study and the limited sample size (*n* = 6 per group), the results should be interpreted cautiously, and a power analysis was not performed. Statistical significance thresholds were established as follows: *p* < 0.01 denoted extremely significant differences between groups, while 0.01 < *p* < 0.05 denoted significant differences. In correlation analysis, an absolute Pearson correlation coefficient exceeding 0.8 was considered to indicate a very strong correlation, values ranging from 0.5 to 0.8 demonstrated a strong correlation, values spanning 0.3 to 0.5 demonstrated a moderate correlation, and values below 0.3 were categorized as representing a weak correlation.

## 3. Results

### 3.1. Meat Quality Traits

Independent-sample *t*-tests were employed to assess differences in LDM meat quality characteristics between the W and S groups. As demonstrated in Table 2, no significant differences were observed between the W and S groups regarding pH 0, pH 24, L*, a*, or b*. Conversely, CL in the S was markedly diminished compared with the W group (*p* = 0.046). Likewise, the SF value in the S group was markedly diminished versus the W group (*p* = 0.026).

### 3.2. Histomorphological Analysis

**Figure 1:**
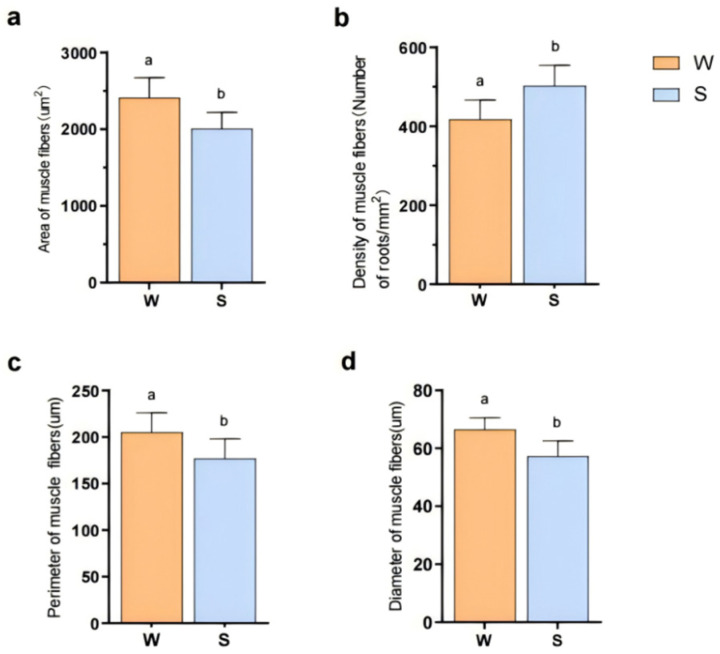
Morphological comparison of LDM fiber cross-sections between the W group and the S group. (**a**): muscle fiber area; (**b**): muscle fiber density; (**c**): muscle fiber perimeter; (**d**): muscle fiber diameter. Different lowercase letters (a, b) above bars indicate significant differences (*p* < 0.05) between the two groups.

**Figure 2:**
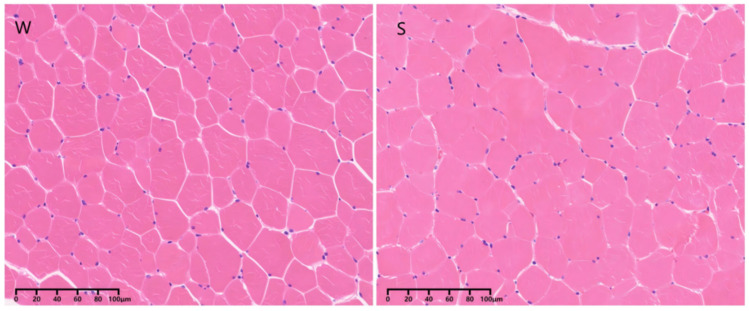
H&E-stained tissue sections.

Cross-sectional slices of LDM fibers from the W and S groups were measured and subjected to comparative analysis. The results demonstrated that, relative to the W group, the S group exhibited a markedly higher LDM fiber density, whereas muscle fiber diameter, perimeter, and cross-sectional area were markedly diminished (Figure 1 and Figure 2).

### 3.3. Transcriptome Sequencing Analysis

#### 3.3.1. Principal Component Analysis (PCA) and Orthogonal Partial Least Squares Discriminant Analysis (OPLS-DA)

**Figure 3:**
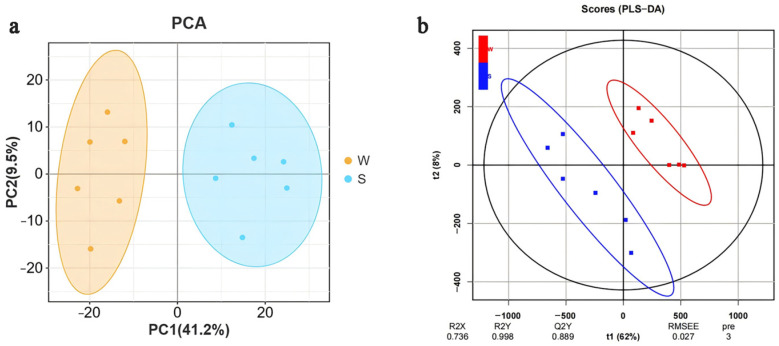
(**a**): PCA plot; (**b**): OPLS-DA plot.

Transcriptome sequencing analysis was conducted on LDM samples obtained from 12 Kazakh horses. PCA findings demonstrated that the initial two principal components represented 50.7% of the total variance, explaining 9.5% and 41.2% of the variance, respectively, as illustrated in Figure 3a. Clear separation was noted between the W and S groups, which formed two distinct and independent clusters. Further analysis using OPLS-DA is shown in Figure 3b, where pronounced differences between the W and S groups were also evident in the OPLS-DA score plot.

#### 3.3.2. Screening and Clustering Analysis of DEGs

**Figure 4:**
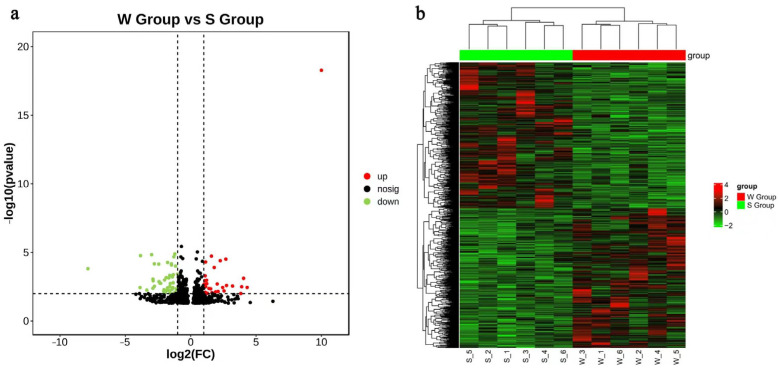
DEGs between the W and S groups. (**a**): Volcano plot of identified genes, including genes from RNA-seq, where green represents downregulation and red represents upregulation; (**b**): clustering heatmap of DEGs.

DEGs in the LDM between the W and S groups were identified using the DESeq2 package in R, with screening criteria defined as *p*-value ≤ 0.05 and |log2 (fold change)| ≥ 0. In total, 848 DEGs were obtained, including 415 markedly upregulated DEGs and 433 markedly downregulated DEGs (Figure 4a). Hierarchical clustering heatmap analysis demonstrated that gene expression profiles exhibited clear clustering within each group, while pronounced differences were evident between the W and S groups (Figure 4b).

#### 3.3.3. GO and KEGG Enrichment Analysis

**Figure 5:**
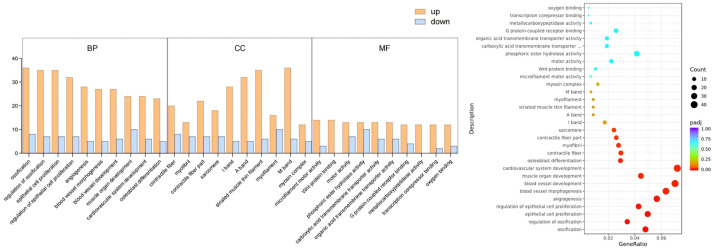
GO enrichment analysis (BP: biological process; CC: cellular component; MF: molecular function).

GO enrichment analysis indicated that DEGs in the LDM between the W and S groups were predominantly enriched in biological process ontology terms linked to skeletal, muscular, and vascular development. Within cellular component ontology terms, enrichment was mainly localized to muscle fiber and muscle fiber contraction-related components. In terms of molecular function, relatively few DEGs were identified, with phosphoesterase hydrolase activity representing the primary enriched category in the gene ontology. However, the difference was not statistically significant (Figure 5).

**Figure 6:**
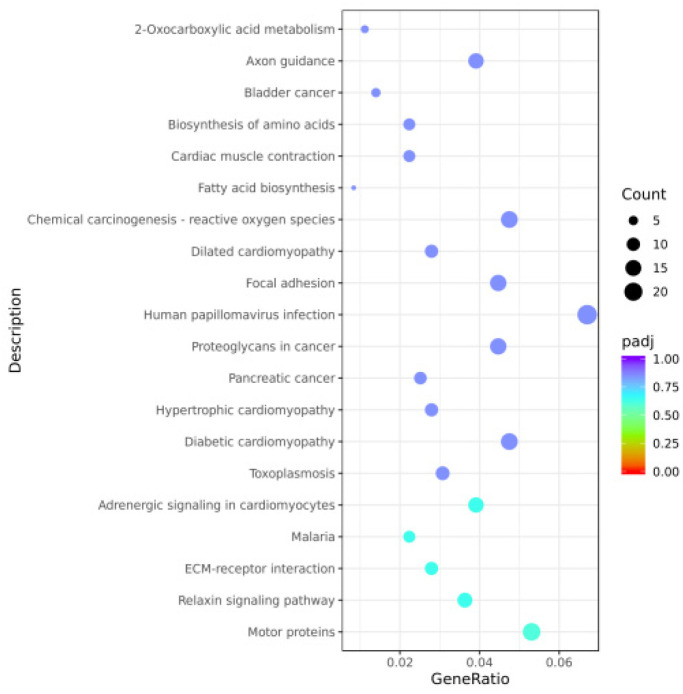
KEGG enrichment analysis.

KEGG pathway enrichment analysis of DEGs demonstrated that, among the top 20 KEGG pathways, 17 pathways exhibited significant differences (0.01 < *p* < 0.05). Notably, the dynein assembly pathway exhibited highly significant enrichment (*p* < 0.01) and presented the highest GeneRatio (Figure 6).

#### 3.3.4. Pearson Correlation Analysis of Phenotypic Traits and Selected Genes’ Expression

**Figure 7:**
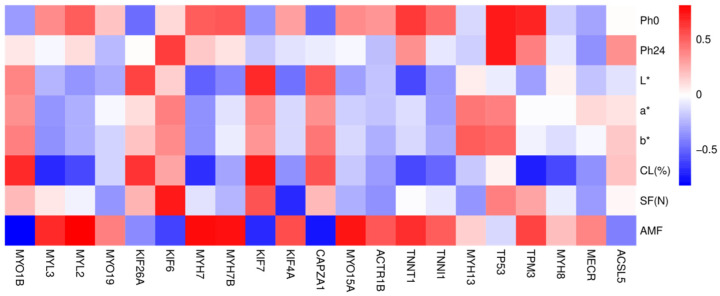
Pearson correlation analysis. L*, lightness; a*, redness-greenness value; b*, yellowness-blueness value.

Pearson correlation analysis was performed to examine the relationships between genes linked to dynein and fatty acid formation within markedly different KEGG pathways and LDM meat quality parameters. The results indicated that MYL3, MYL2, MYH7, MYH7B, MYO15A, TNNT11, TNNI1, and TPM3 exhibited strong positive correlations with area of muscle fiber (AMF) and varying degrees of negative correlations with SF and CL. Among these genes, KIF4A showed a strong negative correlation with SF, whereas TPM3 demonstrated a strong negative correlation with CL. In contrast, MYO1B, KIF7, and CAPZA1 displayed strong negative correlations with AMF and varying degrees of positive correlations with SF and CL, with KIF7 exhibiting a strong positive correlation with CL (Figure 7). The specific correlation coefficients are presented in Supplementary Table S1.

#### 3.3.5. Validation of Sequencing Data Accuracy by qRT-PCR

**Figure 8:**
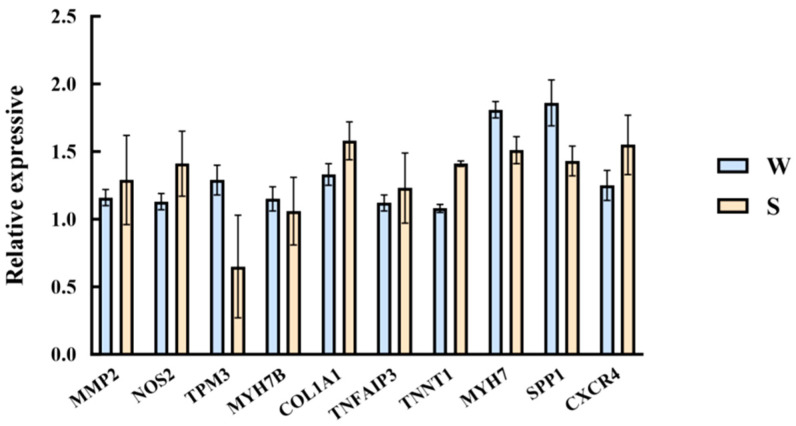
qRT-PCR validation of DEGs.

To verify the accuracy of transcriptome sequencing results in this experiment, 10 DEGs (MMP2, NOS2, TPM3, MYH7B, COL1A1, TNFAIP3, TNNT1, MYH7, SPP1, and CXCR4) were arbitrarily chosen for qRT-PCR validation. The RNA samples used for fluorescence-based quantitative validation were identical to those applied for RNA-seq library construction and sequencing. As illustrated in Figure 8, the expression levels and overall trends of the 10 validated genes determined by qRT-PCR showed consistency with the expression patterns obtained from RNA-seq analysis, suggesting that the RNA-seq data generated in this experiment were reliable.

## 4. Discussion

This study compared differences in meat quality indicators and muscle fiber morphological characteristics between stallions and geldings. Transcriptomic variations in the longissimus dorsi muscle were analyzed for the first time, and key candidate genes were identified. The findings provide fundamental data for revealing the mechanism underlying castration-mediated meat quality formation in Kazakh horses.

Muscle quality traits constitute primary determinants of meat market competitiveness, directly influencing market value, consumer acceptance, and final sales volume. Therefore, the identification and improvement of meat quality characteristics that correspond to market demand are essential for promoting the commercialization of the horse meat industry. The findings of this experiment demonstrated that surgical castration exerted no significant effects on pH values or meat color parameters (L*, a*, b*) in horse meat, which aligns closely with findings reported by Kaic et al. [19], indicating that sex-related factors exert minimal influence on the physicochemical properties of horse meat. However, other studies have reported that stallions exhibit brighter meat color than geldings [20]. Although discrepancies remain regarding the effects of surgical castration on meat color and pH, notable improvements linked to surgical castration were observed in key eating quality traits, particularly CL and tenderness. These results indicated that, relative to the W group, the S group displayed a markedly lower CL (CL: (15.43 ± 2.88)%) vs. (11.40 ± 3.21), accompanied by a significant reduction in SF (SF: (99.84 ± 4.38) N) vs. (96.21 ± 4.36). This may be due to differences in breed, age, or slaughter conditions. A decrease in SF represents a direct indicator of enhanced meat tenderness, suggesting that surgical castration can effectively improve the palatability of horse meat. Consistent with these findings, Razmaitė et al. [21] reported no significant differences in pH between stallions and geldings, while meat from geldings exhibited greater tenderness than that from stallions. Nevertheless, in terms of meat color, stallions were shown to possess brighter meat color compared with geldings. Overall, surgical castration markedly diminished CL and SF in the LDM of Kazakh horses, thereby enhancing meat tenderness and increasing juiciness after cooking, which aligns well with current market preferences for high-quality horse meat.

Morphological observation of muscle tissue sections is regarded as a fundamental technical approach for elucidating the formation mechanisms of meat quality traits and for the precise regulation of meat quality. H&E staining, as a classical method for basic histomorphological assessment, constitutes an important analytical tool for examining the structural features and morphological characteristics of muscle fibers. The tenderness properties of horse meat are closely related to muscle fiber cross-sectional area and density indices. Previous studies have demonstrated that the cross-sectional areas of three muscle fiber types in castrated cattle were markedly smaller than those observed in intact bulls [22]. Similarly, experiments conducted by Rossetti et al. reported that multiple muscles in mice exhibited markedly reduced muscle fiber cross-sectional areas at 7 weeks following castration [23]. The findings of this experiment indicated that surgical castration markedly diminished muscle fiber diameter in horses (*p* < 0.05), while muscle fiber density displayed a marked increasing trend (*p* < 0.05), which is consistent with previously reported results. Collectively, these findings indicate that surgical castration can markedly decrease muscle fiber cross-sectional area while increasing muscle fiber density.

With recent advances in RNA sequencing technologies, transcriptome analysis has been widely employed to identify and characterize key genes associated with meat quality traits. In livestock species such as pigs, cattle, and sheep, numerous studies have demonstrated that castration alters hormone levels, which in turn regulate the expression of genes involved in muscle development, fat deposition, and extracellular matrix (ECM) remodeling. These molecular changes ultimately influence meat tenderness, flavor, and other important quality traits. Wang et al. [24] found in Huainan male pigs that castration induced 935 differentially expressed genes (DEGs) in the longissimus dorsi muscle, involving pathways of hormone secretion, lipid metabolism, and muscle development. Sun et al. [25] reported in Holstein cattle that castration significantly modified the expression of genes related to intramuscular fat deposition, improving meat tenderness and flavor. Zhang et al. [26] demonstrated in sheep that castration enhanced meat performance by regulating genes involved in ECM remodeling and lipid metabolism. In equine species, Razmaitė et al. [21] showed in adult horses that castrated males had significantly higher meat tenderness and intramuscular fat content than intact males; Wubulikasimu et al. [2] conducted transcriptomic studies on Kazakh horse muscles and found that the longissimus dorsi muscle (LDM) exhibited the most significant gene expression differences related to meat quality, with age and sex regulating gene expression through ECM–receptor interaction and muscle fiber development pathways. In the present study, a total of 848 statistically significant DEGs were identified, which may influence the muscle quality of castrated Kazakh horses through coordinated regulation involving both gene activation and repression.

To further explore the molecular basis of the observed phenotypic changes, GO and KEGG enrichment analyses were conducted on the identified DEGs, revealing the major biological processes and signaling pathways associated with castration-mediated regulation of meat quality traits. With the rapid development of transcriptomic technologies, RNA sequencing (RNA-Seq) has become an effective approach for elucidating the molecular mechanisms underlying castration-induced alterations in skeletal muscle gene expression. Wubulikasimu et al. [2] found that castration significantly altered the transcription levels of genes related to muscle fiber development and ECM remodeling in the longissimus dorsi muscle of Kazakh horses. Razmaitė et al. [21] reported distinct expression patterns of genes involved in lipid metabolism and myocyte differentiation between intact and castrated adult horses. GO enrichment results revealed that the DEGs were significantly enriched in biological processes, including skeletal development, muscle development and blood vessel development. At the cellular component level, enriched terms were mainly associated with muscle fibers and contraction-related components, indicating that these genes maintain the structural stability of muscle tissue. Phosphoric ester hydrolase activity dominated the molecular function enrichment, suggesting that DEGs modulate muscle development by regulating intracellular phosphorylation signaling, energy metabolism and myocyte differentiation. KEGG pathway analysis demonstrated that DEGs affected phenotypic variation of longissimus dorsi muscle via dynein formation, relaxin signaling pathway and ECM–receptor interaction. Previous studies have proven that the dynein pathway serves as a critical regulatory pathway in muscle biology. As motor proteins in the cortical pulling pathway, dyneins govern myonuclear positioning, which is essential for normal muscle fiber structure and function [23,27]. The relaxin signaling pathway regulates muscle development by mediating myocyte differentiation and ECM remodeling. ECM–receptor interaction facilitates material exchange and signal transduction between myocytes and the surrounding microenvironment, closely linked to muscle fiber assembly and functional maintenance [28,29]. In conclusion, these DEGs collaboratively regulate multiple physiological processes, such as myocyte constitution, substance transport, cytoskeleton organization, muscle fiber assembly and material exchange between cells and the extracellular microenvironment, thereby accounting for the developmental differences in longissimus dorsi muscle between stallions and geldings.

Pearson correlation analysis (Figure 8; Supplementary Table S1) provided functional links between DEG expression levels and meat quality parameters. We identified 11 core candidate genes that showed strong correlations (|r| > 0.8) with CL, SF, or muscle fiber area. Previous investigations have demonstrated that the expression ratio of MYL3 can function as a potential molecular marker for the evaluation of muscle toughness [30]. The intact sequence of the MYL2 gene is essential for maintaining normal muscle contractile function and stabilizing muscle fiber types. Sequence mutations may indirectly exert adverse effects on meat quality-related traits by altering the basal physiological state of muscle [30,31]. Alterations in the MYH7 gene expression are strongly linked to muscle fiber growth, and differential expression of this gene further affects muscle fiber type composition and histological characteristics, thereby contributing to the regulation of muscle structure [32,33]. Additionally, MYH7 and MYH7B have been reported to participate in the myosin microRNA regulatory network by encoding mmu-mir-208 and mmu-mir-499, respectively, which are involved in regulating myofiber-related genes [34]. MYO15A is linked to intramuscular fat deposition and meat tenderness, and its regulatory effects may be linked to the coordinated development of muscle cells and adipocytes [35]. TNNT1, a characteristic gene of slow skeletal muscle, influences meat quality by participating in muscle fiber type transformation [36]. Lin et al. [37] reported that TNNI1, together with TNNT1 and other genes, forms a gene network related to the troponin complex and is involved in skeletal muscle regulation. Furthermore, Zhang et al. [38] demonstrated that elevated expression of TNNI1 is linked to key meat quality traits, including beef drip loss and meat color. Through synergistic interactions with myofiber structure-related genes, TNNI1 contributes to differences in tenderness, juiciness, and other quality attributes across anatomical locations by modulating myofiber stability and contractile properties. TPM3 has been shown to influence skeletal muscle growth by regulating myoblast proliferation and differentiation, thereby providing a molecular basis for the modulation of meat quality traits such as tenderness and muscle fullness [39]. MYO1B may also be indirectly linked to meat quality traits, including AMF deposition and meat color, through its effects on muscle fiber growth rate and structural integrity.

Collectively, these findings demonstrated that dynein-related pathways constituted the primary mechanisms through which surgical castration influenced LDM meat quality. These pathways contributed to meat quality variation by modulating muscle fiber contraction, growth and development, and fiber type transformation. The coordinated expression and interaction of these DEGs led to distinct differences in LDM meat quality between the W and S groups, thereby providing a useful reference for subsequent evaluations of meat quality.

Several limitations should be acknowledged. First, the sample size (n = 6 per group) is modest, and a power analysis was not performed; thus, the results should be considered exploratory. Second, we only examined the longissimus dorsi muscle; castration may exert different effects on other muscles (e.g., biceps femoris or gluteus medius). Finally, we did not measure serum testosterone levels, which would have provided a direct link between the endocrine status and gene expression changes.

## 5. Conclusions

This study demonstrates that surgical castration of three-year-old Kazakh horses significantly improves longissimus dorsi meat tenderness and water-holding capacity (reduced SF and CL) without altering color or pH. These improvements are associated with a smaller myofiber cross-sectional area and a coordinated downregulation of contractile genes (e.g., MYL2, MYL3, MYH7, TNNI1, TPM3) enriched in the dynein assembly pathway. Our findings provide the first transcriptomic resource for castrated horse muscle and highlight candidate genes for future marker-assisted management of horse meat quality. Given the exploratory nature of this study (n = 6 per group), independent validation in larger cohorts is warranted.

## Figures and Tables

**Table 1:** Primer information.

Gene Name	Primer Sequence
*GAPDH*	F: TTGCCCTCAACGACCACTTT R: TCTTGCTGGGTGATTGGTGG
*MMP2*	F: ATGGCGCCCATTTACACGTA R: AGCTCTTGAATGCCCTTGATG
*NOS2*	F: GCCAAGGTCTGAGCTACCTG R: GAGTGCCTGGCTGAGTGAG
*TPM3*	F: CTTGGAGCGCACAGAGGAAC R: GATCCAGAACAGAGCAGAAAC
*MYH7*	F: TGAGAAGGGCAAAGGCAAG R: ATGATGCAACGCACGAAG
*COL1A1*	F: TGCGAAGACACCAAGAACTG R: GACTCCTGTGGTTTGGTCGT
*TNFAIP3*	F: GAAGAGCAGCTGAGGTCGAG R: AGGATGTTCTTGCAGGAGGTG
*TNNT1*	F: CGTAAAAAGCCTCTGAACATCG R: ATACAGCACGTTGATCTCGTAT
*MYH7B*	F: GAGGACCAAGTATGAGACAGAC R: GTCTTCTCAAGAGACGAACACT
*SPP1*	F: TGGCGAGTTTGAGAAGGTGT R: TTTGCAAGGCCCGATGTAGT
*CXCR4*	F: AGCAAAGTGACTCCGAGGAC R: GTGTATATACGGAACCCGTCCA

**Table 2:** Comparison of meat quality traits in LDM between W and S groups.

	Group W	Group S	*p*-Value
pH0	5.84 ± 0.28	5.86 ± 0.17	0.738
pH24	5.52 ± 0.26	5.41 ± 0.10	0.102
L*	27.54 ± 1.94	26.45 ± 1.28	0.056
a*	22.94 ± 4.42	21.42 ± 4.50	0.315
b*	13.01 ± 1.98	11.97 ± 2.79	0.141
CL(%)	15.43 ± 2.88	11.40 ± 3.21	0.046
SF(N)	99.84 ± 4.38	96.21 ± 4.36	0.026

L*, lightness; a*, redness-greenness value; b*, yellowness-blueness value.

## Data Availability

The transcriptomic dataset generated in this study is available in an online repository. The names of the repository/repositories and accession number(s) can be found below: Sequence Read Archive (PRJNA1455212).

## Supplementary Materials

The following supporting information can be downloaded at: https://www.mdpi.com/article/10.3390/biology15120959/s1, Table S1: Pearson Correlation Analysis Data.

## Abbreviations

The following abbreviations are used in this manuscript:

LDM	Left Longissimus Dorsi Muscle
DEGs	Differentially Expressed Genes
CL	Cooking Loss
SF	Shear Force
AMF	Area of Muscle Fiber
GO	Gene Ontology
KEGG	Kyoto Encyclopedia of Genes and Genomes
AS	Alternative splicing
qRT-PCR	Quantitative real-time polymerase chain reaction
pH 0	Muscle pH value at 0 h post-slaughter
pH 24	Muscle pH value at 24 h post-slaughter
